# Ca^2+^/Calmodulin and Apo-Calmodulin Both Bind to and Enhance the Tyrosine Kinase Activity of c-Src

**DOI:** 10.1371/journal.pone.0128783

**Published:** 2015-06-09

**Authors:** Silviya R. Stateva, Valentina Salas, Estefanía Anguita, Gustavo Benaim, Antonio Villalobo

**Affiliations:** 1 Department of Cancer Biology, Instituto de Investigaciones Biomédicas, Consejo Superior de Investigaciones Científicas and Universidad Autónoma de Madrid, c/ Arturo Duperier 4, E-28029, Madrid, Spain; 2 Instituto de Biología Experimental, Facultad de Ciencias, Universidad Central de Venezuela, Caracas, Venezuela; 3 Instituto de Estudios Avanzados (IDEA), Caracas, Venezuela; Hungarian Academy of Sciences, HUNGARY

## Abstract

Src family non-receptor tyrosine kinases play a prominent role in multiple cellular processes, including: cell proliferation, differentiation, cell survival, stress response, and cell adhesion and migration, among others. And when deregulated by mutations, overexpression, and/or the arrival of faulty incoming signals, its hyperactivity contributes to the development of hematological and solid tumors. c-Src is a prototypical member of this family of kinases, which is highly regulated by a set of phosphorylation events. Other factor contributing to the regulation of Src activity appears to be mediated by the Ca^2+^ signal generated in cells by different effectors, where the Ca^2+^-receptor protein calmodulin (CaM) plays a key role. In this report we demonstrate that CaM directly interacts with Src in both Ca^2+^-dependent and Ca^2+^-independent manners *in vitro* and in living cells, and that the CaM antagonist *N*-(6-aminohexyl)-5-chloro-1-naphthalenesulfonamide (W-7) inhibits the activation of this kinase induced by the upstream activation of the epidermal growth factor receptor (EGFR), in human carcinoma epidermoide A431 cells, and by hydrogen peroxide-induced oxidative stress, in both A431 cells and human breast adenocarcinoma SK-BR-3 cells. Furthermore, we show that the Ca^2+^/CaM complex strongly activates the auto-phosphorylation and tyrosine kinase activity of c-Src toward exogenous substrates, but most relevantly and for the first time, we demonstrate that Ca^2+^-free CaM (apo-CaM) exerts a far higher activatory action on Src auto-phosphorylation and kinase activity toward exogenous substrates than the one exerted by the Ca^2+^/CaM complex. This suggests that a transient increase in the cytosolic concentration of free Ca^2+^ is not an absolute requirement for CaM-mediated activation of Src in living cells, and that a direct regulation of Src by apo-CaM could be inferred.

## Introduction

Calmodulin (CaM) is a versatile Ca^2+^ receptor protein present in all so far tested eukaryotic cells that binds to and modulates the function of hundreds of proteins with or without enzymatic activity, exerting therefore multiple physiological roles in the cell (reviewed in [1]). Thus, CaM couples the Ca^2+^ signal generated by multiple effectors (e.g. hormones and growth factors) to appropriate cellular responses. Nevertheless, its Ca^2+^-free form (apo-CaM) also binds to and regulates a variety of target proteins (reviewed in [2]). A diversity of mechanisms for the interaction of CaM with many of their targets has been described (reviewed in [3–5]). Albeit there is not an universal consensus sequence, CaM binding sites present in target proteins are classified either as amphipathic basic α-helical domains with conserved bulky hydrophobic residues at positions 1-(5)-10, 1-(8)-14 or 1–16; IQ motifs with the general sequence (FILV)Qxxx(RK)Gxxx(RK)xx(FILVWY), where x represents any amino acid; or IQ-like motifs with the general sequence (FILV)Qxxx(RK)xxxxxxxx [6–10].

Among the multiple physiological functions of CaM, its role in the control of a variety of systems implicated in cell proliferation and other important cellular processes relevant for the biology of tumor cells, such as programmed cell death and autophagy, has been amply studied (reviewed in [11]). Our group has been studying the action of CaM on the control of receptor tyrosine kinases (RTKs) implicated in cell proliferation. Among these, important mediators in the proliferative response of cells are the erythroblastic leukemia viral oncogene homologue (ErbB) family members (ErbB1/EGFR/HER1, ErbB2/HER2/neu, ErbB3/HER3 and ErbB4/HER4) that play a prominent role in cancerogenesis. We demonstrated the Ca^2+^-dependent interaction of CaM with the cytosolic juxtamembrane segment (residues 645–660) of the epidermal growth factor receptor (EGFR) and its action in contributing to the ligand-dependent activation of the receptor [12–14]. This occurs most likely by a mechanism proposed by McLaughlin and collaborators [15], in which Ca^2+^/CaM helps to release the auto-inhibition of the ligand-free EGFR mediated by the electrostatic interaction of the positively charged CaM-binding site (and part of the tyrosine kinase domain) with the negatively charged inner leaflet of the plasma membrane. Likewise, we also demonstrated a Ca^2+^-dependent binding of CaM to ErbB2 and its regulatory effects on the activation of the receptor and downstream signaling pathways [16]. Other RTKs are also known to bind CaM, including: the insulin receptor, where CaM regulates its activity [17]; and TrkA, where CaM appears to control its proteolytic processing [18].

Non-receptor tyrosine kinases belonging to the Src family also play prominent roles in proliferation and cancerogenesis (reviewed in [19]). Several members of the Src kinase family are known to phosphorylate CaM (reviewed in [20]), underscoring that CaM interacts with the catalytic site of these kinases. In addition to this, and most importantly, CaM interacts with some regulatory site(s) present in Src. Thus, an earlier report demonstrated that a myristoylated peptide corresponding to the N-terminal of v-Src interacts with the Ca^2+^/CaM complex, while the non-myristoylated peptide was not able to do so [21]. Also, it has been proposed that upon Ca^2+^ entry in effector-stimulated cells, CaM residing in lipid rafts interacts in a Ca^2+^-dependent manner with and activates c-Src, and in turn c-Src phosphorylates and inhibits protein phosphatase 2A (PP2A), preventing in this manner its inhibitory action on Akt and henceforth promoting melanoma tumor growth [22]. The activation of c-Src by Ca^2+^/CaM was also demonstrated in transfected neuroblastoma cells overexpressing α-synuclein, a cytotoxic protein abundant in Lewy bodies in Parkinson’s disease, by a mechanism also implicating PP2A [23]. The direct interaction of CaM with a recombinant glutathione S-transferase (GST)-Src fusion protein was demonstrated to occur via a dual Ca^2+^-dependent and Ca^2+^-independent mechanism, although mutation of the proposed CaM-binding site located at the Src homology domain 2 (SH2) of c-Src only partially prevented CaM binding [24]. Interestingly, Src was shown to co-immunoprecipitate with CaM but not with tyrosine-phosphorylated CaM in keratinocytes [25]. Also, the unique and SH3 domains of c-Src has been shown to bind acidic lipids, usually present in the inner leaflet of the plasma membrane, and binding of Ca^2+^/CaM to the unique domain has been proposed to regulate this process [26].

Nevertheless, these reports did not fully clarify the actual mechanism by which CaM interacts and activates c-Src, or if this process always occurs in the cell in a Ca^2+^-dependent manner. This highlights the need for additional work to determine whether CaM controls the tyrosine kinase activity of c-Src in both Ca^2+^-dependent and/or Ca^2+^-independent manners. In this report we demonstrated, using an *in vitro* assay system and in living tumor cells, that CaM directly binds to c-Src in both Ca^2+^-dependent and Ca^2+^-independent manners, and that Ca^2+^/CaM and apo-CaM both enhances the tyrosine kinase activity of c-Src.

## Materials and Methods

### Reagents

Radiolabelled [γ-^32^P]ATP (triethylammonium salt) (3,000 Ci/mmol) (1 Ci = 37 GBq), Hyperfilm-MP x-ray films, calmodulin-Sepharose 4B, and the enhanced chemiluminescence (ECL) kits were obtained from GE Healthcare-Amersham. The Pierce Classic Magnetic IP/Co-IP kit was obtained from Thermo Scientific. ATP (sodium salt), L-glutamic acid and L-tyrosine polymer (poly-L-(Glu:Tyr)) (4:1), Sepharore 4B, rabbit polyclonal anti-phospho-Src (Y418) (recognizing human phospho-Y416), and anti-mouse (Fc specific) immunoglobulin G (IgG) polyclonal (goat) antibody coupled to horseradish peroxidase were purchased from Sigma-Aldrich. The polyvinylidene difluoride (PVDF) membranes were obtained from Pall Corporation. Rabbit monoclonal anti-Src (human) (clone 36D10, isotype IgG), rabbit polyclonal anti-phospho-Src family (Y416) and rabbit monoclonal anti-glyceraldehyde-3-phosphate dehydrogenase (GAPDH) (clone 14C10, isotype IgG) antibodies were obtained from Cell Signaling Co. Goat anti-rabbit IgG (H+L) polyclonal antibody coupled to horseradish peroxidase was from Life Technologies. Mouse monoclonal anti-phospho-tyrosine antibody (clone 4G10, isotype IgG2bκ), and active (763 U/mg) purified 6His-tagged full-length recombinant human c-Src expressed by baculovirus in Sf21 insect cells were purchased from Millipore. One unit of Src activity corresponds to the incorporation of 1 nmol of phosphate into 250 μM cdc2 substrate peptide per min at 30°C using 100 μM ATP according to the manufacture’s datasheet.

### Cell culture

Human epidermoid carcinoma A431 cells (ATCC CRL-1555) and human breast adenocarcinoma SK-BR-3 cells (ATCC HTB-30) were obtained from the American Type Culture Collection (ATCC), and grown in Dulbecco’s modified Eagle’s medium (DMEM) supplemented with 10% (v/v) fetal bovine serum (FBS), 2 mM L-glutamine and 40 μg/ml gentamicin at 37°C in an humidified air atmosphere containing 5% CO_2_.

### Expression and purification of calmodulin

The expression and purification of wild type CaM, CaM(Y99D/Y138D) and CaM(Y99E/Y138E) from transformed *Escherichia coli* BL21(DE3)pLysS was done using protocols previously described [27, 28].

### Preparation of the cell membrane fraction

A431 cells were washed with PBS (137 mM NaCl, 2.7 mM KCl, 12 mM Na/K-phosphate, pH 7.4), gently scraped from the plates, harvested by centrifugation, and lysed by mechanical disruption using a homogenizer in 3 ml of an ice-cold hypotonic buffer containing 15 mM Hepes-Na (pH 7.4), 1 mM ethylene glycol-bis(2-aminoethylether)-*N*,*N*,*N′*,*N′*-tetraacetic acid (EGTA), and a cocktail of protease inhibitors containing: 0.5 mM 4-(2-aminoethyl) benzenesulfonyl fluoride (AEBSF), 0.4 μM aprotinin, 25 μM bestatin, 7.5 μM [1-[*N*-[(L-3-trans-carboxyoxirane-2-carbonyl)-L-leucyl]amino]-4-guanidinobutane] (E-64), 10 μM leupeptin, 5 μM pepstatin A, and freshly prepared 0.6 mM phenylmethylsulfonyl fluoride (PMSF). The lysate was incubated 10 min on ice and centrifuged at 130,000 g for 30 min at 4°C. The supernatant was discarded, and the pellet resuspended in the same buffer and centrifuged as above. This pellet, that corresponds to the membrane fraction, was washed with the same buffer but without EGTA, centrifuged again, resuspended in 3 ml of 25 mM Hepes-Na (pH 7.4) containing the protease inhibitors and stored at -80°C for later use.

### Polyacrylamide gel electrophoresis and Western blot

Proteins were separated by polyacrylamide gel electrophoresis in the presence of sodium dodecyl sulfate (SDS-PAGE) in a 5–20% (w/v) linear gradient slab gel of polyacrylamide and 0.1% (w/v) sodium dodecyl sulfate (pH 8.3) at 6–10 mA overnight [29]. The proteins were electrotransferred from the gels to PVDF or nitrocellulose membranes for 2 h at 300 mA in a medium containing 48 mM Tris-base, 36.6 mM L-glycine, 0.04% (w/v) sodium dodecyl sulfate (SDS), and 20% (v/v) methanol. In the case of CaM, a nitrocellulose membrane instead of PVDF membrane was regularly used. The proteins were fixed with 0.2% (v/v) glutaraldehyde in 25 mM Tris-HCl (pH 8), 150 mM NaCl and 2.7 mM KCl (TNK buffer) for 45 min, and transiently stained with Fast Green to ascertain the regularity of the transfer procedure. The PVDF membranes were blocked with 5% (w/v) bovine serum albumin or 3–5% (w/v) fat-free powdered milk, following the instructions of the antibodies' manufacturers, in 0.1% (w/v) Tween-20, 100 mM Tris-HCl (pH 8.8), 500 mM NaCl and 0.25 mM KCl (T-TBS buffer); and probed overnight at 4°C using a 1/2000 dilution of the corresponding primary antibody, and for 1 h at room temperature using a 1/5000 dilution of the appropriate secondary anti-IgG antibody coupled to horseradish peroxidase. The bands were visualized upon development with ECL, following instructions from the manufacturer, and exposure of x-ray films for appropriate periods of time. The intensity of the bands was quantified using the ImageJ 1.46r program (National Institutes of Health, USA).

### Isolation of Src by CaM-affinity chromatography and immobilized-CaM pull-down

Cell membrane-anchored Src from A431 cells was detached from the membrane fraction upon incubation with 1% (w/v) Triton X-100 and 5% (w/v) glycerol at 0°C for 10 min. The sample was centrifuged at 130,000 g for 30 min and the supernatant was used to isolate Src by CaM-affinity chromatography or pull-down using immobilized CaM. The isolation of Src by Ca^2+^-dependent CaM-affinity chromatography was carried out loading the solubilized membrane fraction in a small column (1–2 ml bed volume) of CaM-Sepharose 4B equilibrated with a buffer containing 25 mM Hepes-NaOH (pH 7.4), 1% (w/v) Triton X-100, 5% (w/v) glycerol, 0.1 mM CaCl_2_, and the protease inhibitors cocktail described above (Ca^2+^-buffer). The column was washed with 25 volumes of the Ca^2+^-buffer, and Src was eluted using the same buffer but containing 1 mM EGTA instead of CaCl_2_ (EGTA-buffer). Proteins in the eluted fractions (0.6 ml) were precipitated with 10% (w/v) trichloroacetic acid and processed by SDS-PAGE and Western blot for Src identification. The pull-down of Src was done using a slurry of CaM-Sepharose 4B beads (200 μl) in a buffer containing 25 mM Na-Hepes (pH 7.4), 1% (w/v) Triton X-100 and 5% (w/v) glycerol supplemented either with 0.1 mM CaCl_2_ or 1 mM EGTA. We used naked Sepharose 4B beads as negative binding control. The beads were washed 10 times with the corresponding Ca^2+^ or EGTA buffers before processing the samples by SDS-PAGE and Western blot for Src identification as described above.

### Co-immunoprecipitation of CaM with Src

Immunoprecipitation of Src from a detergent-solubilized cell extract of A431 cells (2 mg protein) was performed using the Pierce Classic Magnetic IP/Co-IP kit, an anti-Src antibody and protein-A/G following the manufacture instructions. The samples were processed for SDS-PAGE and Western blot using anti-CaM and anti-Src antibodies as described above.

### Src phosphorylation assay

The auto-phosphorylation of recombinant c-Src (0.1 μg) was assayed at 37°C in a medium containing 15 mM Tris-HCl (pH 7.5), 5 mM MgCl_2_, 1 mM dithiothreitol, 1 mM EGTA, 1.2 mM CaCl_2_ (when added), and 2.3 μM wild type CaM, or the phospho-mimetic CaM(Y99D/Y138D) and CaM(Y99E/Y138E) mutants, when added. The reaction was started upon addition of 2 mM ATP and stopped by the addition of Laemmli buffer and boiled for 5 min. The samples were subjected to Western blot and probed with an anti-phospho-tyrosine antibody or an anti-phospho-Y416-Src specific antibody to detect active phosphorylated Src, and an anti-Src (total) antibody and/or GAPDH antibody as loading controls. The phosphorylation of the artificial exogenous substrate poly-L-(Glu-Tyr) (0.1 mg/ml) by recombinant c-Src (1 unit) was carried out at 37°C during 15 min in a medium containing 15 mM Hepes-NaOH (pH 7.4), 5 mM MgCl_2_, 1 mM dithiothreitol, 1 mM EGTA, 1.1 mM CaCl_2_ (when added) and 4 μM CaM (when added) using 10 μM (2 μCi) [γ-^32^P]ATP as substrate. The ^32^P-labeled poly-L-(Glu-Tyr) and ^32^P-labeled c-Src were visualized by autoradiography.

### Src activation assays in living cells

In the case of A431 cells, Src was activated either upon ligand-dependent activation of the EGFR, as an upstream signaling component of c-Src [30], adding 10 nM epidermal growth factor (EGF) during 2 min or upon induction of oxidative stress by adding 1 mM H_2_O_2_ for 15 min, as described [31, 32]. In the case of SK-BR-3 cells, upon induction of oxidative stress by adding 1 mM H_2_O_2_ for 15 min, as above. The reaction was arrested upon addition of ice-cold 1% (w/v) trichloroacetic acid, and the samples were processed by SDS-PAGE and Western blot. The PVDF membranes were probed with anti-phospho-tyrosine or anti-phospho-Y416-Src specific antibodies to detect active phosphorylated Src, anti-phospho-tyrosine to detect activated EGFR, and anti-Src (total), anti-EGFR (total) and/or anti-GAPDH antibodies as loading controls.

### Statistical analysis

Two-tailed Student’s t test was performed using the Microsoft Excel (Microsoft Co., Redmon, WA) or GraphPad Prism (GraphPad Software Inc., La Jolla, CA) software programs. Data were expressed as the mean ± SEM and differences were considered significant at p ≤ 0.05 as indicated in the legends to the figures.

## Results

### Ca^2+^-dependent and Ca^2+^-independent interaction of CaM with Src

**Fig 1:**
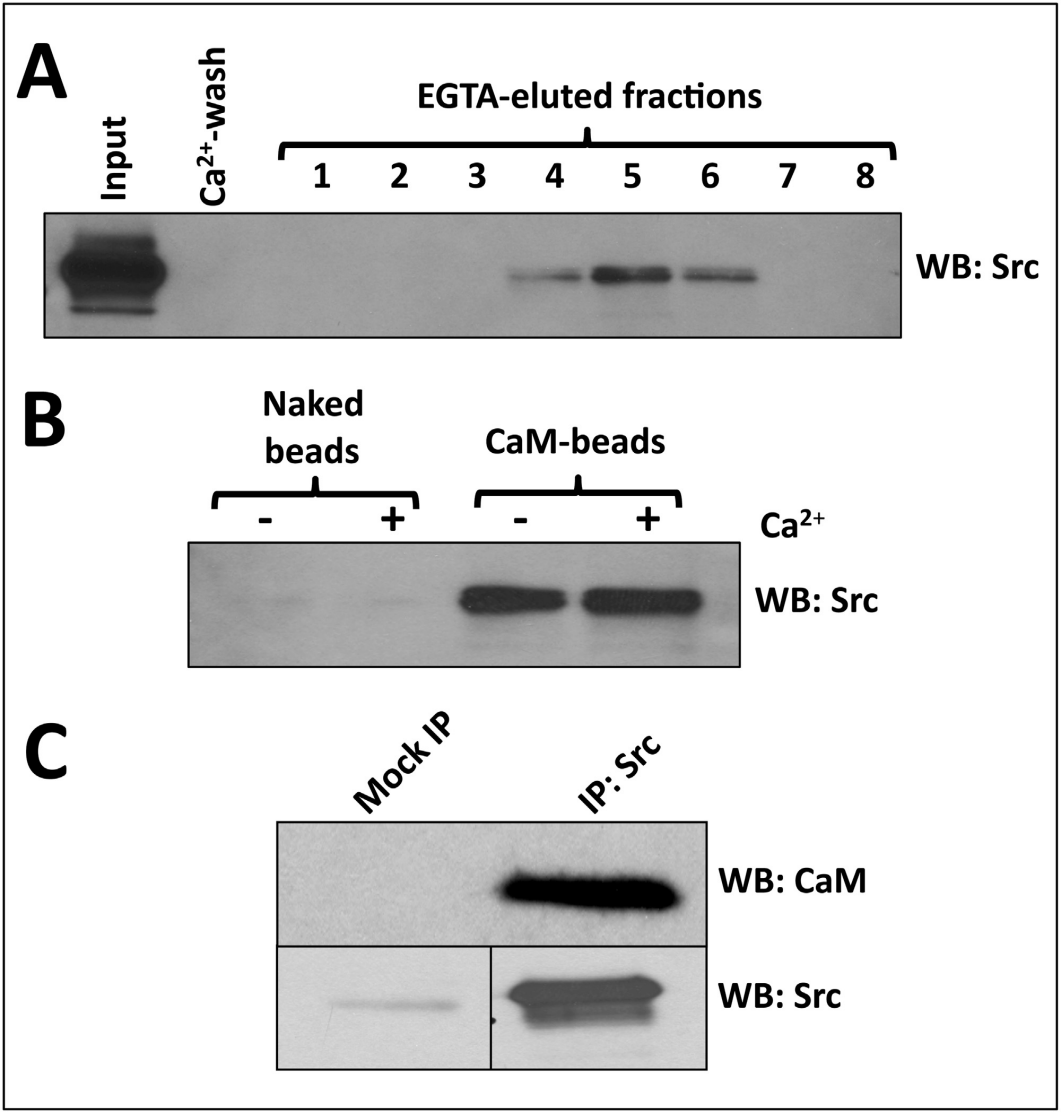
Calmodulin directly interacts with Src in the absence and presence of Ca^2+^. (*A*) Ca^2+^-dependent calmodulin-affinity chromatography of Src solubilized from the membrane fraction of A431 cells was performed as described in Materials and Methods. The material loaded in the CaM-Sepharose column (*input*), the last fraction after washing with the Ca^2+^-buffer (*Ca* ^*2+*^ *-wash*), and the EGTA-eluted fractions were analyzed by Western blot using an anti-Src antibody. (*B*) Pull-down of Src solubilized from a membrane fraction of A431 cells was performed in the absence (-) and presence (+) of Ca^2+^ using CaM-Sepharose (*CaM-beads*) as described in Materials and Methods. Naked Sepharose 4B beads were used as negative binding control. The presence of bound Src to the beads was analyzed by Western blot using an anti-Src antibody. (*C*) Src was solubilized from a membrane fraction of A431 cells (2 mg protein) and immunoprecipitated (IP) using an anti-Src antibody as described in Materials and Methods. The immunocomplex was processed by Western blot using anti-CaM and anti-Src antibodies. Non-relevant rabbit IgG (~22 μg) was used as a negative control (mock IP).

We first performed Ca^2+^-dependent CaM-affinity chromatography of a detergent-solubilized membrane fraction from A431 tumor cells in order to determine whether Src binds CaM in a Ca^2+^-dependent manner. Fig 1A shows that indeed at least a fraction of the Src loaded in the CaM-Sepharose column in the presence of Ca^2+^ can be eluted with a buffer containing the Ca^2+^-chelating agent EGTA. As this technique does not show whether part of Src remained bound to CaM-Sepharose in the absence of Ca^2+^ (presence of EGTA), we performed pull-down experiments using immobilized CaM in the absence and presence of Ca^2+^. Fig 1B shows that Src binds to CaM covalently conjugated to the Sepharose beads, both in the presence of Ca^2+^ and its absence (presence of EGTA), while no significant binding of Src was detected using CaM-free naked beads. Furthermore, we demonstrated that CaM co-immunoprecipitated with Src solubilized from A431 cells (Fig 1C). Overall, these experiments show direct interaction between CaM and Src and that this interaction occurs by both Ca^2+^-dependent and Ca^2+^-independent mechanisms, in agreement with previous findings [24].

### The calmodulin antagonist W-7 inhibits Src activation in living cells

**Fig 2:**
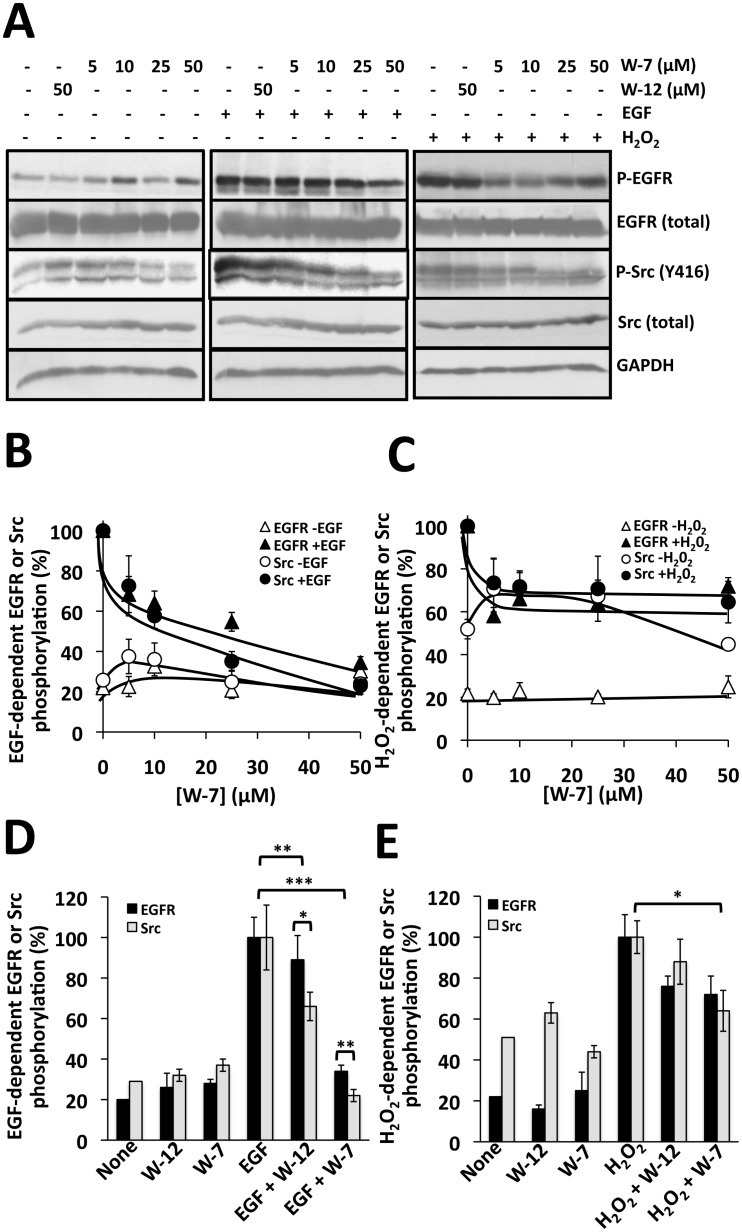
W-7 inhibits EGFR- and H_2_O_2_-mediated Src activation in A431 cells. (*A*) A431 cells were incubated in the absence (-) and presence (+) of the indicated concentrations of W-12 or W-7 during 15 min. Thereafter, the cells were stimulated either with 10 nM EGF for 2 min or 1 mM H_2_O_2_ for 15 min. The reaction was arrested and subjected to Western blot analysis as described in Materials and Methods, and probed with anti-phospho-tyrosine (4G10), anti-phospho-Src (Y416), and anti-EGFR (total), anti-Src (total) and anti-GAPDH antibodies as loading controls. (*B*, *C*) The plots present the mean ± SEM EGF-dependent (n = 6) and H_2_O_2_-dependent (n = 3) activation of Src at increasing concentrations of W-7 from experiments similar to those shown in *A*. (*D*, *E*) The plots present the mean ± SEM EGF-dependent (n = 6) and H_2_O_2_-dependent (n = 3) activation of Src in the absence (*None*) and presence of 50 μM W-12 or 50 μM W-7 from experiments similar to those shown in *A*. Statistically significant differences with p < 0.05 (*), p < 0.005 (**) and p < 0.0001 (***) using the Student’s t-test are indicated.

Ligand-dependent activation of EGFR results in the down-stream activation of Src [30]. To determine whether CaM is implicated in EGFR-mediated Src activation in EGFR-overexpressing A431 cells, we performed experiments in the presence of the CaM antagonist *N*-(6-aminohexyl)-5-chloro-1-naphthalenesulfonamide (W-7) and the less potent but not inactive inhibitor *N*-(4-aminobutyl)-2-naphthalenesulfonamide (W-12). Fig 2A (*left and center panels*), 2B and 2D show that activation of the EGFR by its ligand EGF also results in Src phosphorylation at Y416 (activation) as expected, and that increasing concentrations of W-7 progressively inhibit both phosphorylation processes. In contrast, high concentration (50 μM) of W-12, a chlorine-free analogue of W-7 with far lower affinity for CaM (IC_50_ of 260 μM versus 28 μM, respectively, inhibiting Ca^2+^/CaM-dependent phosphodiesterase) [33, 34], has a much lower inhibitory effects on EGFR activation than on Src activation (Fig 2D). We have previously demonstrated that in living cells the Ca^2+^/CaM complex aided the ligand-dependent activation of the EGFR, and that W-7 inhibits this process [12–14]. Nevertheless, as the inhibitory action of W-7 on Src phosphorylation (activation) was significantly stronger than that observed on ligand-dependent EGFR auto(trans)phosphorylation, this suggests a direct activatory effect of CaM on Src. Furthermore, we demonstrated that W-7 not only inhibits the ligand-dependent phosphorylation (activation) of EGFR but the ligand-dependent tyrosine-phosphorylation of many downstream proteins (*not shown*).

Yet, the results discussed above do not represent a clear-cut evidence for a direct action of CaM on Src activation in living cells. Therefore, we searched for another way to activate Src without the involvement of EGFR to test the possible inhibitory effect of W-7. Fig 2A (*right panel*), 2C and 2E show that hydrogen peroxide, a known activator of Src [31, 32], strongly enhances Src auto-phosphorylation (activation) in A431 cells as expected, and that W-7 indeed significantly inhibits this process in contrast to the lower effect of the low-affinity CaM inhibitor W-12 at identical concentrations (Fig 2E). Moreover, hydrogen peroxide strongly activated the EGFR, a process that was inhibited by the Src inhibitor PP1 (*not shown*), suggesting that this was a consequence of EGFR phosphorylation by Src, as this process has been extensively documented [35–39], and it is known that H_2_O_2_ induces EGFR transactivation by a Src-mediated mechanism [40].

**Fig 3:**
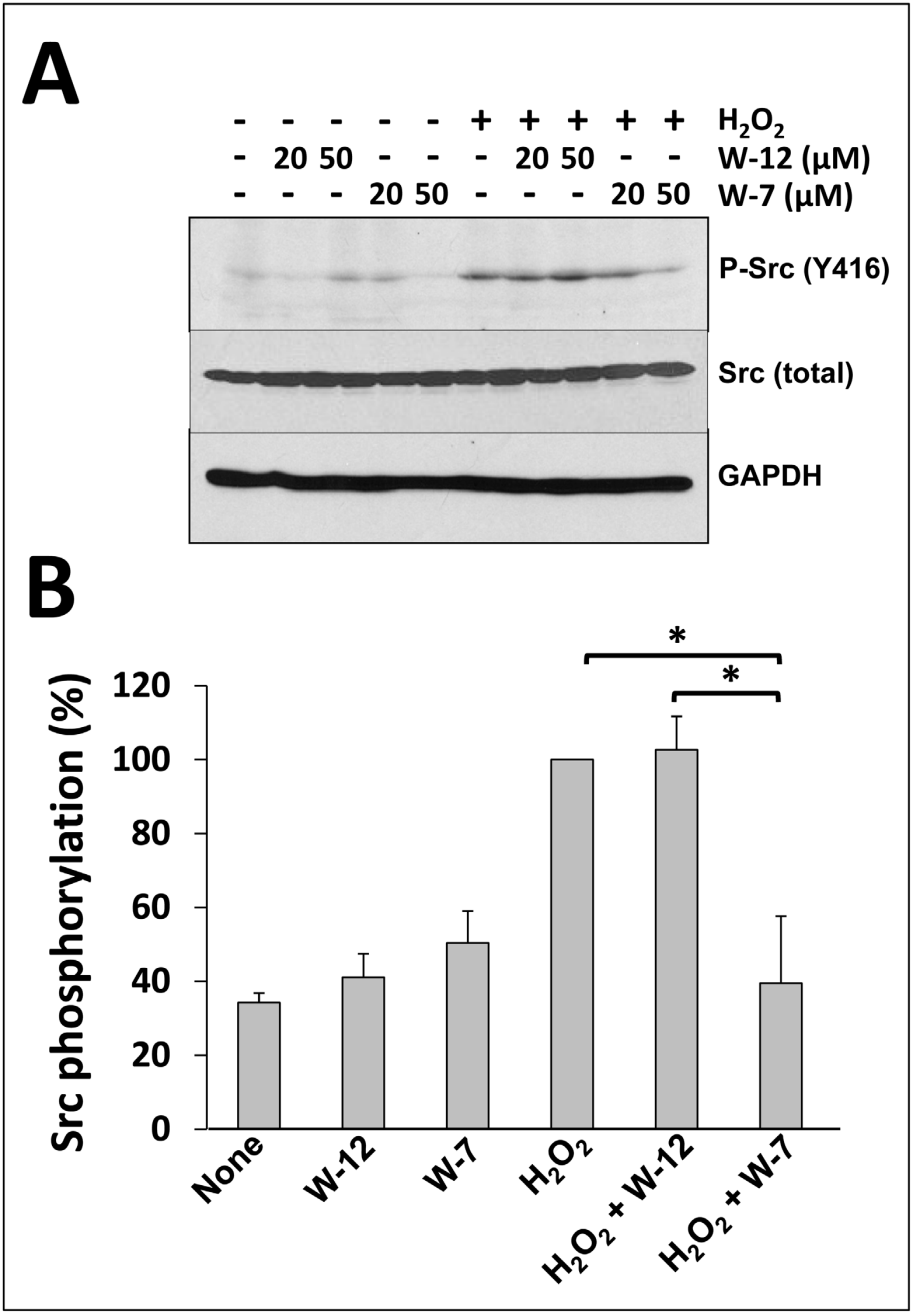
W-7 inhibits H_2_O_2_-mediated Src activation in SK-BR-3 cells. (*A*) SK-BR-3 cells were incubated in the absence (-) and presence (+) of the indicated concentrations of W-12 or W-7 during 15 min. Thereafter, the cells were stimulated with 1 mM H_2_O_2_ for 15 min. The reaction was arrested and subjected to Western blot analysis as described in Materials and Methods, and probed with anti-phospho-Src (Y416), and anti-Src (total) and anti-GAPDH antibodies as loading controls. (*B*) The plot presents the mean ± SEM (n = 5) H_2_O_2_-dependent activation of Src in the absence (*None*) and presence of either 50 μM W-12 or 50 μM W-7 from experiments similar to those shown in *A*. Statistically significant differences with p < 0.05 (*) using the Student’s t-test are indicated.

We also tested the inhibitory action of W-7 on hydrogen peroxide-dependent activation of Src in a different cell line. Fig 3A and 3B show that hydrogen peroxide enhances the phosphorylation (activation) of Src in SK-BR-3 cells, and that the CaM antagonist W-7 inhibits the hydrogen peroxide-dependent activation of Src while W-12 does not. As W-7 appears to inhibit CaM in its Ca^2+^-bound form [41] and may not affect apo-CaM, these results clearly suggest that Ca^2+^/CaM regulates Src activity in living cells, although no information can be extracted from these experiments on the potential control that apo-CaM may exert on Src activation.

### Both Ca^2+^/CaM and apo-CaM activate Src

**Fig 4:**
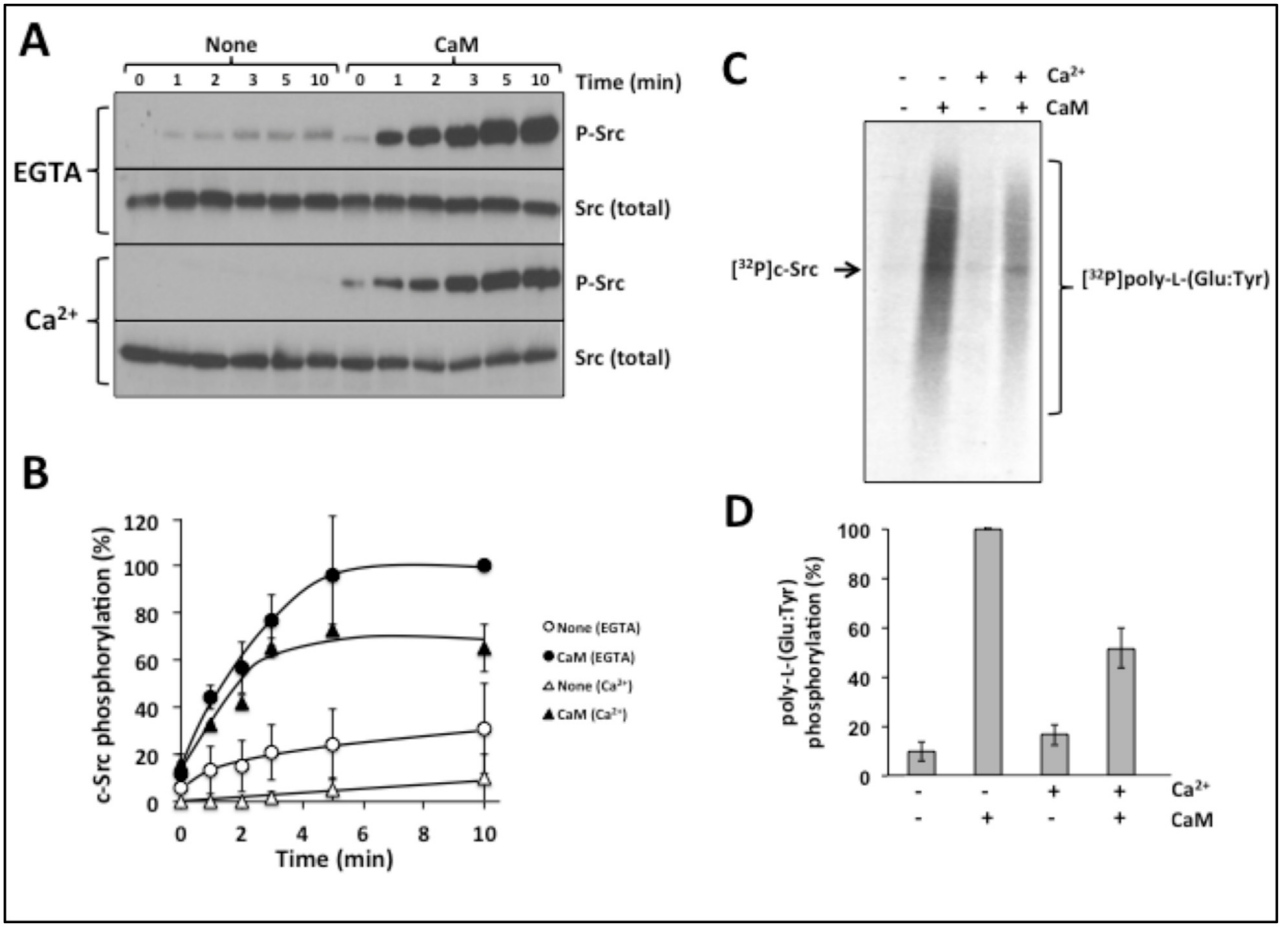
Apo-calmodulin and Ca^2+^/calmodulin both activate c-Src. (*A*) The auto-phosphorylation assay of recombinant c-Src was performed in the absence and presence of CaM and in the absence and presence of Ca^2+^ for the indicated time as described in Materials and Methods. The samples were subjected to Western blot and probed with an anti-phospho-tyrosine antibody. Duplicate samples were probed with an anti-Src (total) antibody as loading control. (*B*) The plot presents the mean ± range c-Src phosphorylation in the absence (*None*) and presence of CaM, and in the absence (*EGTA*) and presence of Ca^2+^ from two independent experiments similar to the one shown in *A*. (*C*) Phosphorylation of poly-L-(Glu:Tyr) by recombinant c-Src was performed using [γ-^32^P]ATP as substrate as described in Material and Methods. [^32^P]-labeled c-Src (*arrow*) and [^32^P]-labeled poly-L-(Glu:Tyr) (*bracket*) are indicated. (*D*) The plot presents the mean ± SEM poly-L-(Glu:Tyr) phosphorylation in the absence and presence of 4 μM CaM and 100 μM CaCl_2_ (Ca^2+^) from three independent experiments similar to the one shown in *C*.

Significant evidence has been obtained on the activatory action of Ca^2+^/CaM on the tyrosine kinase activity of Src [22–24]. However, it was assumed that this process is always preceded by the generation of a Ca^2+^ signal upon cell activation by a variety of effectors required for the formation of the Ca^2+^/CaM complex. Little is known, however, on the possible action of apo-CaM on the activation of Src. To solve this question, we tested the effect of CaM on the auto-phosphorylation (activation) of human c-Src in the absence and presence of Ca^2+^ using a full-length recombinant protein. We ascertained that the recombinant c-Src used was indeed full-length without truncation of the N-terminal, as the commercial supplier provided the sequence, the purification of the protein was performed using the 6His-tag located in the N-terminal, and the molecular mass perfectly matched the expected 60 kDa in SDS-PAGE. Fig 4A and 4B show that little auto-phosphorylation of c-Src was observed in the absence of CaM and that the presence of this modulator strongly enhances its auto-phosphorylation in both conditions. The activation of c-Src was significantly stronger in the absence of Ca^2+^ (presence of EGTA) than in its presence. And surprisingly, this effect was also observed when the residual basal activity of c-Src was assayed in the absence of CaM. This suggests a direct inhibitory action of calcium ion on the kinase.

To ascertain that the activatory action of CaM on Src was not limited to its auto-phosphorylation, but also enhances its tyrosine kinase activity toward exogenous substrates, we tested the phosphorylation of the artificial substrate poly-L-(Glu:Tyr). Fig 4C and 4D show that indeed the phosphorylation of poly-L-(Glu:Tyr) by recombinant Src was strongly enhanced by the presence of CaM, both in the absence and presence of Ca^2+^. And again it was in the former condition when CaM exerted its maximum action on Src activation.

**Fig 5:**
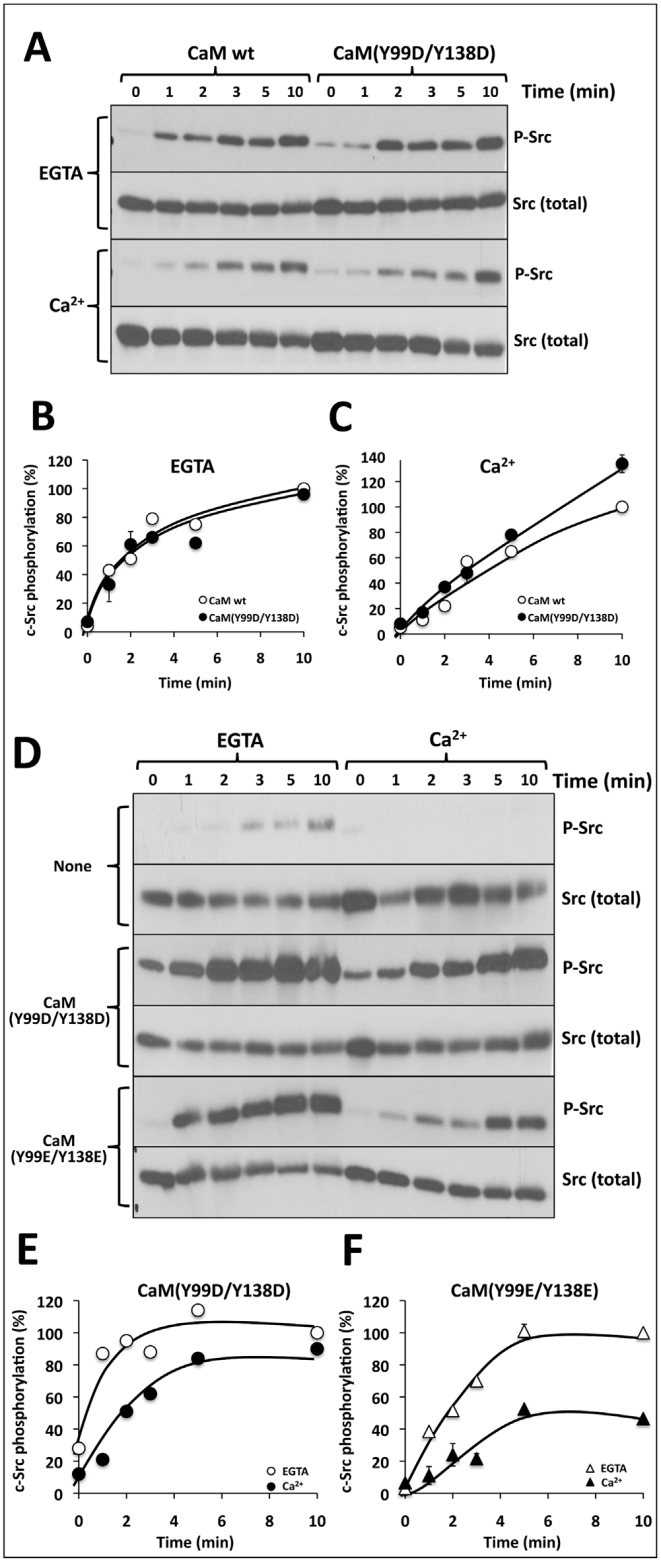
Phospho-(Y)-mimetic CaM mutants also activate c-Src. (*A*) Auto-phosphorylation assays of recombinant c-Src (0.1 μg) was performed in a medium containing 15 mM Tris-HCl (pH 7.5), 5 mM MgCl_2_, 1 mM dithiothreitol, 1 mM EGTA and where indicated 1.2 mM CaCl_2_ (200 μM free Ca^2+^) in the presence of either wild type CaM or CaM(Y99D/Y138D) in a total volume of 100 μl. The samples were processed for SDS-PAGE and Western blot using anti-phospho-tyrosine and anti-Src antibodies as described in Materials and Methods. (*B*, *C*) The plots present the mean ± SEM (n = 2) c-Src phosphorylation in the presence of wild type CaM or CaM(Y99D/Y138D) in the absence and presence of free Ca^2+^ from experiments similar to the ones shown in *A*. (*D*) Auto-phosphorylation assays of recombinant c-Src (0.1 μg) was performed in a medium containing 15 mM Tris-HCl (pH 7.5), 5 mM MgCl_2_, 1 mM dithiothreitol, 1 mM EGTA and where indicated 1.2 mM CaCl_2_ (200 μM free Ca^2+^) in the absence or presence of either CaM(Y99D/Y138D) or CaM(Y99E/Y138E) in a total volume of 100 μl. The samples were processed for SDS-PAGE and Western blot using anti-phospho-tyrosine and anti-Src antibodies as described in Materials and Methods (*E*, *F*). The plots present the mean ± SEM c-Src phosphorylation in the presence of CaM(Y99D/Y138D) (n = 1) or CaM(Y99E/Y138E) (n = 2), and in the absence and presence of free Ca^2+^ from experiments similar to the ones shown in *A*.

As CaM is known to be phosphorylated by different Src family kinases (reviewed in [20]), we tested the action of phospho-(Y)-mimetic CaM mutants, on the auto-phosphorylation (activation) of recombinant c-Src. Fig 5A–5C show little, if any, significant differences in the activatory action of wild type CaM versus CaM(Y99D/Y138D) both in the absence and presence of Ca^2+^. Moreover, both CaM(Y99D/Y138D) and CaM(Y99E/Y138E) had the capacity to activate c-Src more strongly in the absence of Ca^2+^ (presence of EGTA) than in its presence (Fig 5D–5F). This effect was similar to the one exerted by wild type CaM (see Fig 2A and 2B).

## Discussion

In this report we show that Src not only binds CaM in the absence and presence of Ca^2+^, as others have previously demonstrated [24], but that the Ca^2+^/CaM complex, and more efficiently apo-CaM, both enhance the auto-phosphorylation and catalytic activity of Src toward exogenous substrates. Although the activation of Src by different signals appears to require Ca^2+^, as demonstrated by the attenuation of Src activation in cells loaded with the Ca^2+^-chelator [1,2-bis(2-aminophenoxy)ethane-*N*,*N*,*N′*,*N′*-tetraacetic acid tetrakis(acetoxymethyl ester)] (BAPTA-AM) [42, 43], the Ca^2+^-independent action of CaM on Src activation advises for a partial revision of previous findings on the necessary involvement of an executor Ca^2+^ signal preceding the binding of CaM to Src [22, 23, 42, 43]. The drastic reduction of the cytosolic concentration of free Ca^2+^ imposed in BAPTA-loaded cells may also affect Ca^2+^/CaM-dependent systems upstream of Src, as for example the EGFR [12–14], and not exclusively Src activation. Therefore, a transient Ca^2+^ rise may not be an obligatory step to attain activation of Src since apo-CaM exerts this role even more effectively than Ca^2+^/CaM. One possibility, however, could be that oscillations in the cytosolic concentration of free Ca^2+^ indeed could act as a rate modulator of the activity of the Src/CaM complex, where CaM could be already tethered to Src, as it occurs in other CaM-binding proteins such as distinct Ca^2+^-channels [44]. Thus, the kinase activity in the Src/CaM complex could decrease when the concentration of Ca^2+^ rises, by occupying the Ca^2+^-binding sites of tethered CaM, and conversely increases when the concentration of Ca^2+^ falls and the cation is released from CaM. These events may not necessarily be a simple two-stage cycle (Ca^2+^-free and Ca^2+^-bound steps), as CaM has two high-affinity and two low-affinity Ca^2+^-binding sites [1]. Thus, this process may occur as a multi-stage cycle where a variable number of Ca^2+^-binding sites are free or occupied at a given time, what may result in a variable rate of Src activity and a more fine-tuned regulation of the kinase.

An alternative possibility is that distinct Ca^2+^-dependent and Ca^2+^-independent CaM-binding sites could exist in Src. Yuan and collaborators [24] proposed as the CaM-binding site of human Src the sequence ^203^KHYKIRKLDSGGF^215^ (residues 204–214 in the GST-tagged protein), located in the terminal part of the SH2 domain. However, mutation of this site substituting the sequence KHYKIRKLDS by alanine residues only partially prevented CaM binding [24]. This suggests the existence of additional CaM-binding sites in Src. We identified *in silico* in human c-Src two additional potential CaM-binding sites, that we denote atypical IQ-like motifs, corresponding to the sequences: ^146^ **IQ**AEEWYFG**K**IT**R** ^158^, located in the proximal region of the SH2 domain, and ^311^ **LQ**EAQVM**KK**L**R** ^321^, located in the proximal region of the tyrosine kinase domain. These sites may contribute to the binding of apo-CaM, as many IQ- and related IQ-like motifs are known to be receptor sites for Ca^2+^-free CaM in different target proteins [2, 6].

The CaM antagonist W-7 is known to interact with phospholipids. In fact, we have demonstrated that both W-7/W-13 efficiently prevent the binding of a peptide corresponding to the CaM-BD of EGFR (residues 645–660) to lipid vesicles [45]. This opens the possibility that the action of W-7 on Src activity could be mediated at least in part by disturbing the known interaction of the unique domain of the kinase with the inner leaflet of the plasma membrane [26]. We have observed that W-7 slightly increases in a biphasic manner the basal activity of Src in non-stimulated cells (Fig 2B and 2C), similar to what we observed with W-13 activating the EGFR in the absence of ligand [45]. However, the inhibitory effect of W-7 on Src activation induced by EGF or H_2_O_2_ addition indicates that this effect is mainly due to CaM inhibition. W-7 has been widely used in living cells to antagonize CaM and the effects that this inhibition exerts in a variety of CaM-dependent systems. Nevertheless, we cannot exclude off-target direct effect of W-7 on c-Src in living cells, as well as in all experimental systems so far studies. Particularly, when this CaM antagonist has been shown to inhibit Ca^2+^-dependent protein kinase and to a lesser extent cAMP/cGMP-dependent protein kinases [46].

The non-receptor tyrosine kinase Src is subjected to complex regulatory mechanisms mediated by phosphorylation events that control its activation status [19, 47–49]. The stabilization of its activation loop induced by auto-phosphorylation of Y416 maintains the kinase in an open conformation, allowing substrate binding and hence subsequent signal transmission by the resulting phosphorylated substrates [48]. On the other hand, the specific phosphorylation of its C-terminal tail at Y527 by C-terminal Src kinase (Csk), maintains Src in a closed conformation repressing its activity [47]. The identification of Src as a CaM-binding protein, and the proposed location of the CaM-binding site(s) at the SH2 and/or tyrosine kinase domains, suggests that CaM may mediate its action maintaining Src in its open activated conformation.

We also observed that Ca^2+^ *per se* (absence of CaM) has a direct inhibitory action on Src auto-phosphorylation. This is unlikely to be mediated by an exogenous Ca^2+^-dependent system, as for example protein kinase C (PKC), because we used a purified preparation of recombinant Src and the assay was devoid of cofactors required for PKC activation. Although speculative, an interesting possibility is to search for potential EF-hand Ca^2+^-binding pocket(s) in Src.

Src-family kinases including c-Src are known to phosphorylate CaM (reviewed in [20]) as also demonstrated by us [27, 28]. It has been shown in keratinocytes that phospho-(Y138)-CaM does not co-immunoprecipitate with Src, while non-phosphorylated CaM does [25]. Our results using CaM(Y99D/Y138D) and CaM(Y99E/Y138E) suggest that diphospho-(Y99/Y138)-CaM may be able to interact with Src, in contrast to what it was reported with monophospho-(Y138)-CaM [25]. No information, however, is available on the possible action of distinct phospho-(Tyr)-CaM species on Src activity. Nevertheless, the effect of distinct phosphorylated tyrosine residues and/or variable phosphorylation stoichiometry in the regulation of Src should be investigated, as the importance of these parameters has been demonstrated in other CaM-dependent systems (reviewed in [20]).

Another point of interest is the fact that the two partners in the well-known bidirectional activatory trans-phosphorylation between EGFR and Src, where Src phosphorylates the EGFR [35–39], and the EGFR phosphorylates Src [30], are regulated by CaM, albeit in different manners. The regulation of EGFR by CaM is a Ca^2+^-dependent process [12–14], while the regulation of Src by CaM appears to be a two-faced process, Ca^2+^-dependent and Ca^2+^-independent (this work). This opens the door to explore in future work how physiological oscillations in the cytosolic concentration of free Ca^2+^, upon cell stimulation by mitogenic and/or other factors, modifies this mechanism by affecting in different manner both interconnected partners, the EGFR and Src, and hence the proliferative response.
